# CPAP failure in the management of preterm neonates with respiratory distress syndrome where surfactant is scarce. A prospective observational study

**DOI:** 10.1186/s12887-023-04038-6

**Published:** 2023-05-03

**Authors:** Yaser Abdallah, Martha Mkony, Mariam Noorani, Robert Moshiro, Mohamed Bakari, Karim Manji

**Affiliations:** 1 Department of Paediatrics and Child Health, Aga Khan University, P.o Box 38129 Dar es Salaam, Tanzania; 2 Department of Paediatrics and Child Health, Muhimbili National Hospital and Muhimbili University of Health and Allied Sciences, Dar es Salaam, Tanzania; 3 Department of Paediatrics and Child Health, Makerere University College of Health Sciences, Kampala, Uganda

**Keywords:** CPAP failure, Preterm Neonates, Outcomes

## Abstract

**Background:**

The mainstay in the management of preterm neonates with respiratory distress syndrome (RDS) include early Continuous Positive Airway Pressure (CPAP), timely surfactant replacement and mechanical ventilation. Preterm neonates with RDS who fail CPAP are at higher risk for chronic lung disease as well as death. Unfortunately, in low resource settings CPAP may be the only treatment available for these neonates.

**Objective:**

To determine the prevalence of CPAP failure among premature newborns with RDS and associated factors.

**Methods:**

We conducted a prospective observational study over the first 72 h of life on 174 preterm newborns with RDS receiving CPAP at Muhimbili National Hospital (MNH). At MNH newborns with Silverman Andersen Score (SAS) of ≥ 3 are commenced on CPAP; surfactant and mechanical ventilation are very scarce. Study newborns not maintaining oxygen saturation > 90% or with SAS score ≥ 6 despite being on 50% oxygen and PEEP of 6 cmH_2_O and those with > 2 episodes of apnoea needing stimulation or positive pressure ventilation in 24 h were considered as CPAP failure. The prevalence of CPAP failure was determined as a percentage and factors associated were determined by logistic regression. A p-value of < 0.05 was considered significant and 95% confidence interval was used.

**Results:**

Of the enrolled newborns, 48% were male and 91.4% were in-born. The mean gestational age and weight were 29 weeks (range 24–34 weeks) and 1157.7 g (range 800–1500 g) respectively. Of the mothers 44 (25%) received antenatal corticosteroids. Overall CPAP failure was 37.4% and among those weighing ≤ 1200g, it was 44.1% . Most failure occurred within the first 24 h. No factor was identified to be independently associated with CPAP failure. Mortality among those who failed CPAP was 33.8% and 12.8% among those who did not.

**Conclusions:**

In resource limited settings like ours with low up take of antenatal corticosteroids and scarce surfactant replacement a significant portion of preterm neonates especially those weighing ≤ 1200 g with RDS fail CPAP therapy.

## Background

Complications of prematurity account for a significant proportion of the under 5 mortality globally [1]. The majority of these preterm babies are delivered in low- and middle-income countries (LMICs) [2]. Tanzania is among the countries with high preterm birth rates hence addressing the causes of death in this population is crucial in reducing the childhood mortality rates for Tanzania as well as the world at large.

One of the first complications preterm neonates encounter is respiratory distress syndrome (RDS) from lack of surfactant [3]. RDS causes hyaline membrane formation in the lungs making the lungs stiff and poor for gaseous exchange. This disease in the first 3 days of life increases the work of breathing and hypoxia and if not addressed it causes respiratory failure and/or death. Beyond the 3 days some surviving neonates start making natural surfactant and recovery may ensue [4].

The incidence of RDS is inversely related to the gestational age [5]. Although the use of antenatal corticosteroids (ANCs) has been shown to reduce the incidence of RDS; in low- and middle-income countries (LMIC’s) this strategy is underutilized [6]. At Muhimbili National Hospital; Tanzania’s national referral hospital, only 31% of mothers of preterm infants received ANC’s [7].

The mainstay in the management of RDS involves early Continuous Positive Airway Pressure (CPAP), surfactant replacement and mechanical ventilation if needed [8–10]; such therapies are scarce in LMIC’s. Although early use of CPAP among preterm neonates with RDS has been shown to reduce the need for surfactant therapy, mechanical ventilation and death [11–14]; in low resource settings early CPAP may not be possible for various reasons.

Despite feasibility of implementing CPAP therapy in LMIC’s, an estimated 20–40% of preterm neonates fail CPAP therapy [15]. Some of the predictors of CPAP therapy failure include lower gestational age, severe RDS, high fraction of inspired oxygen and delay in CPAP initiation [9, 16–18]. Neonates who fail CPAP are at increased risk for pneumothorax, bronchopulmonary dysplasia (BPD) and death [9, 17, 19, 20].

In settings like ours, where antenatal steroid uptake is very low, CPAP initiation is delayed and surfactant is not readily available, CPAP failure rate may be very high. We conducted this study to establish the prevalence of CPAP failure and the factors associated with it among preterm neonates with RDS. This information should guide care and resource allocation for preterm neonates with RDS in such settings.

## Methods

### Study design and study site

This was a prospective observational study carried out at Muhimbili National Hospital (MNH) which is the largest referral hospital in Tanzania and the teaching hospital for Muhimbili University of Health and Allied Sciences (MUHAS). Annually approximately 10,000 babies are admitted at MNH neonatal unit; with prematurity and low birth weight accounting for nearly 40% of all admissions. The unit has preterm, term, surgical, asphyxia and Kangaroo Mother Care sections.

The neonatal ICU and high dependency area have a maximum capacity of 20 babies at any one time. It is managed by 1 consultant, 1 medical officer and 1 resident in general paediatrics. The nurse: patient ratio on average is 1: 3.

The unit has the capacity of providing CPAP therapy to 15 babies, the CPAP brands available include Diamedica and Vayu using RAM prongs. The unit can also provide mechanical ventilation to 4 babies at any given time. Premature babies with RDS are assessed using the Silverman Andersen Score (SAS) and are started on CPAP therapy if the score is ≥ 3. For severe RDS, surfactant is given if it is available and parents can bear the cost, it is never given prophylactically. If the clinical condition worsens, a baby may be intubated and ventilated depending on availability of a ventilator. A neonate may not get the preferred treatment because of scarcity of resources.

Among neonates with RDS on CPAP; care is escalated if neonate is not maintaining saturation > 90% by increasing the fraction of inspired oxygen by 10% every 30 min to maximum of 50%, PEEP is set at a maximum of 6 cmH_2_O. For those needing > 50% or SAS score > 6 surfactant is recommended. Surfactant is administered thought INSURE method (Intubate, Surfactant administered and Extubate back to CPAP). If parents can’t afford surfactant, they are encouraged to commit to pay for it, this results in delay in administration; meanwhile the neonate would be supported by CPAP with fraction of the inspired oxygen increased to try and maintain saturation > 90%.

Neonates on CPAP who fail to maintain saturation > 90% or with SAS score > 6 on 50% inspired oxygen despite receiving surfactant and those with recurrent apnoeas > 2 episodes in 24 h needing positive pressure ventilation are escalated to mechanical ventilation if a ventilator is available.

Continuous positive airway pressure is weaned if the infant maintain saturation > 90% while on < 30% fraction of inspired oxygen and SAS score < 3. PEEP is never lowered. Neonates weaned off CPAP are managed with nasal prong oxygen 0.5-2 l/min to maintain saturation > 90%.

All preterm neonates are empirically started on antibiotics and intravenous fluids. Aminophylline is given to those with apnoea. Blood pressure is not routinely monitored except for babies on mechanical ventilation.

### Study population

Preterm newborns less than 34 weeks of gestational age and with birth weight between 800 and 1500 g inclusive commenced on CPAP within the first 24 h of life for RDS were enrolled. Neonates with multiple congenital abnormalities, those given surfactant before enrollment and those in whom CPAP was interrupted for ≥ 2 h for any reason were excluded. In our unit, all neonates irrespective of gestational age are supported as long as needed care is available.

### Study variables

Independent variables assessed in our study included gestational age, birth weight, sex, place of birth, mode of delivery, maternal antenatal steroids, risk for sepsis, 5 min APGAR score, time to start CPAP therapy and degree of respiratory distress. The outcome variable was successful or failure of CPAP therapy at 72 h of age. Failure was defined if a neonate on CPAP does not maintain oxygen saturation > 90% or has SAS score > 6 despite being on 50% oxygen and PEEP of 6 cm H_2_O or if a neonate develops > 2 episodes of apnoea needing stimulation or positive pressure ventilation in 24 h.

### Sample size estimation

The sample size was calculated using the formula by Kish [21]. A prevalence of CPAP therapy failure of 20% was used as reported from a study by Prashanth for preterm neonates > 27 weeks and < 37 weeks with RDS [22]. This gave a sample size of 246 and assuming 5% for incomplete records, a total sample size of 258 was computed. However, due to budgetary constraints as well as delays in time to start the study because of the COVID-19 pandemic, we only recruited 174 participants over a 1-year period.

### Study procedure

Data was collected between July 2020 to July 2021. Preterm neonates < 24 h old admitted to the unit between 8 am and 3 pm from Monday to Friday were screened for eligibility. Those who met inclusion criteria were reviewed by a trained research assistant. Research assistants were trained in gestational age estimation using the new Ballard score (NBS) [23], respiratory distress score using Silver Andersen Score (SAS) [24] and how to fill in the data sheet. Baseline information was obtained and anthropometry measured. All observations including the SAS was done hourly for the first 6 h and then 6 hourly for 72 h. The study endpoint was 72 h of age. The maximum number of infants recruited per day was two.

### Clinical management

All participants were managed as per the unit’s guidelines which include continuous saturation and heart rate monitoring, daily SAS, random blood sugar if suspecting low levels, temperature measurement every 6 h for neonates in cots and continuous for those on functional warmers.

### Patients and public involvement

Neither parents nor consumers were involved in the designing, conducting or reporting of findings.

### Data management and analysis

Data was entered into Stata and transferred to SPSS version 23 for analysis. Baseline characteristics were summarized in tables, mean was computed for continuous variables and frequencies for categorical variables. The proportion of preterm neonates who failed CPAP therapy was computed as ratio of those who failed CPAP over total commenced on CPAP therapy and followed up for the study duration.

Univariate analysis was conducted to identify factors associated with CPAP failure, all factors with p value of < 0.05 and those considered scientifically associated with failure were included in multivariable logistic regression analysis to identify factors independently associated with CPAP failure. Kaplan Mayer analysis was used to determine time to failure on CPAP.

## Results

**Fig. 1:**
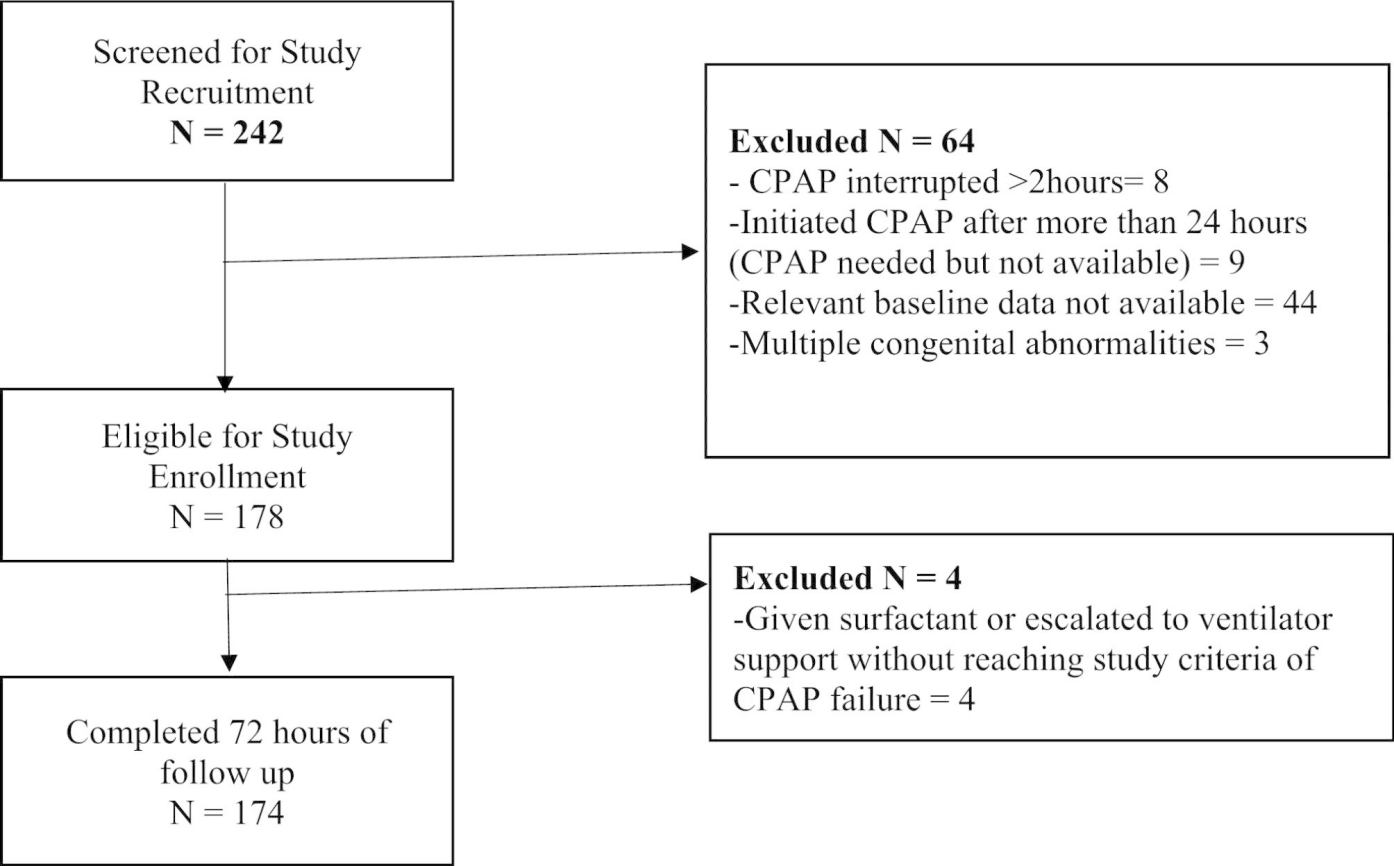
Flow diagram showing the recruitment process

A total of 242 preterm newborns were screened for inclusion into the study and 174 were enrolled and followed up for 72 h. A flow diagram of the recruitment is shown in Fig. 1.

Male infants comprised 48% of the study population, 73.5% of babies were less than 30 weeks gestation and 102 babies (58%) weighed less than 1200 g. Of the study infants 53(30.4%) were small for gestational age (SGA). The majority of the mothers of study infants had not received any antenatal corticosteroids (73.5%), of the 44 mothers who received steroids only 2 of them received the complete 4 doses, 30 received 2 doses and 12 mothers received only 1 dose.

Of the mothers 8 were teenagers, 12 were primiparous, 2 had prolonged rupture of membranes of > 18 h, 31 had hypertension and none of them was diabetic. All study infants were on ampicillin and gentamycin, and none of them was on inotropic support during the study period. A summary of the baseline characteristics is shown in Table 1.

**Table 1:** Baseline characteristics of study participants

Characteristic	Number (%)
**Sex**	
M	81(46.5%)
F	87(50.0%)
Not captured*	6(3.5%)
**Gestational age**	
≤ 30 weeks	125 (71.8%)
> 30 weeks	49 (28.2%)
**Birth weight**	
≤ 1200 g	102(58.6%)
> 1200 g	72(41.4%)
**Small for gestational age (SGA)**	
Yes	53(30.4%)
No	121(69.6%)
**Mode of delivery**	
Vaginal delivery	110(63.2%)
C/s	44 (25.3%)
Not captured*	20 (11.5%)
**Place of birth**	
Inborn	159(91.4%)
Out born	15(8.6%)
**5min APGAR score**	
< 7	48(27.6%)
≥ 7	113(64.5%)
Unknown	13(7.4%)
**Antenatal corticosteroids**	
Yes	44(25.3%)
No	128(73.5%)
Not captured*	2(1.2%)
**Age at initiation of CPAP**	
< 6hrs	95(54.6%)
≥ 6hrs	73(42.0%)
Not captured*	6(3.4%)

*Data not captured by research assistants

The prevalence of CPAP failure was 37.4%. Of the factors studied, only birth weight of ≤ 1200 g was associated with CPAP failure with 44.1% these babies failing CPAP as compared to 27.8% those weighing > 1200 g. The mean time to initiation of CPAP was 6 h and 30 min. The mortality rate of babies in this study was 20.6%. The odds of death was 3 times higher in those who failed CPAP. Table 2 shows factors associated with CPAP failure.

**Table 2:** Factors associated with CPAP failure

Base line Characteristic	CPAP failure	CPAP no failure	OR^a^ (95% CI)	P-value	AOR*	P value
**Sex**						
M F	34(39.1%) 30(37.0%)	53(60.9%) 51(63.0%)	1.090 (0.58–2.03)	0.785	0.902 (0.46–1.73)	
**Gestational age**						
≤ 30 weeks > 30 weeks	48(38.4%) 17(35.0%)	77(61.6%) 32(65.0%)	1.173 (0.58–2.33)	0.649	1.025 (0.48–2.18)	0.949
**Birth weight**						
≤ 1200g > 1200g	45(44.1%) 20(27.8%)	57(55.9%) 52(72.2%)	2.052 (1.07–3.92)	**0.029**	1.933 (0.95–3.91)	0.067
**SGA**						
No Yes	42(34.7%) 23(43.4%)	79(65.3%) 30(56.6%)	0.693 (0.35–1.34)	0.276		
**Mode of delivery**						
C/S SVD	14(31.8%) 50(40.6%)	30(68.2%) 73(59.4%)	0.681 (0.33–1.41)	0.302	0.763 (0.33–1.73)	0.520
**5 min APGAR**						
≤ 7 > 7	17(35.4%) 46(40.7%)	31(64.6%) 67(59.3%)	0.798 (0.39–1.60)	0.529	0.850 (0.42–1.69)	0.643
**CPAP initiation**						
≤ 6hrs > 6hrs	38(40.0%) 26(35.6%)	57(60.0%) 47(64.7%)	1.205 (0.64–2.26)	0.562	1.418 (0.72–2.79)	0.312
**Antenatal steroids**						
Not received Received	51(40.0%) 17(38.6%)	77(60.0%) 27(61.4%)	1.052 (0.52–2.12)	0.887	1.419 (0.66–3.05)	0.369

*AOR for CPAP failure, comparing the first data row to the second, generated from multivariate analysis were computed for variables with p value < 0.05 and all those considered scientifically plausible

^a^OR were regenerated during univariate analysis

NB: For all variables the second raw is the reference

**Fig. 2:**
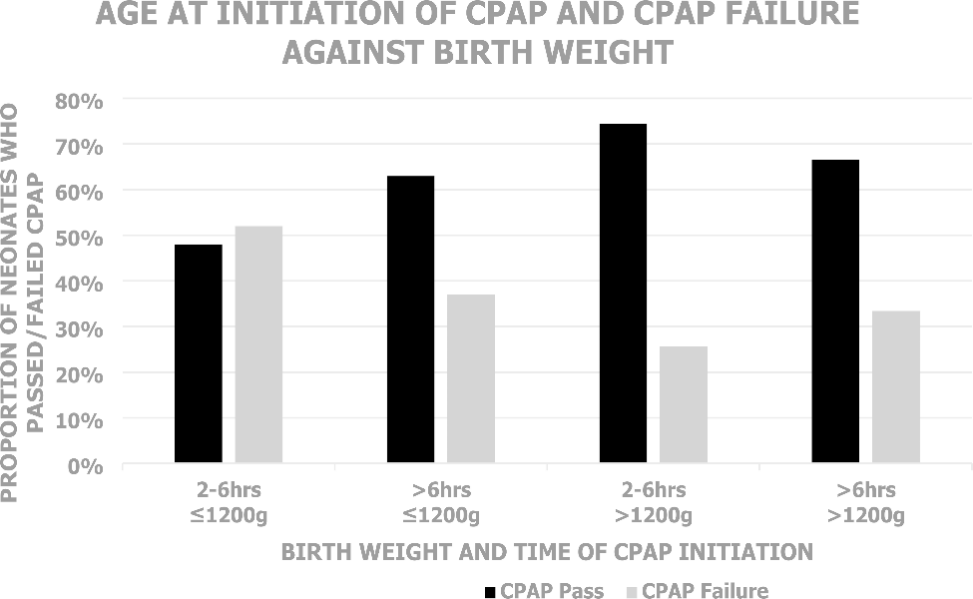
Histogram of age at initiation of CPAP and CPAP failure against Birth weight

Whereas preterm neonates who weighed ≤ 1200 g with RDS had an overall higher CPAP failure rate; we noted that in this group those who needed CPAP within the first 6 h of life were more likely to fail 27/52(51.9%%) as compared to those who needed CPAP at > 6 h 17/46 (36.9%) (Fig. 2). CPAP failure among neonates weighing > 1200 g did not show a difference based on time of initiation. Figure 2 shows age at initiation of CPAP therapy and CPAP failure against birth weight.

**Fig. 3:**
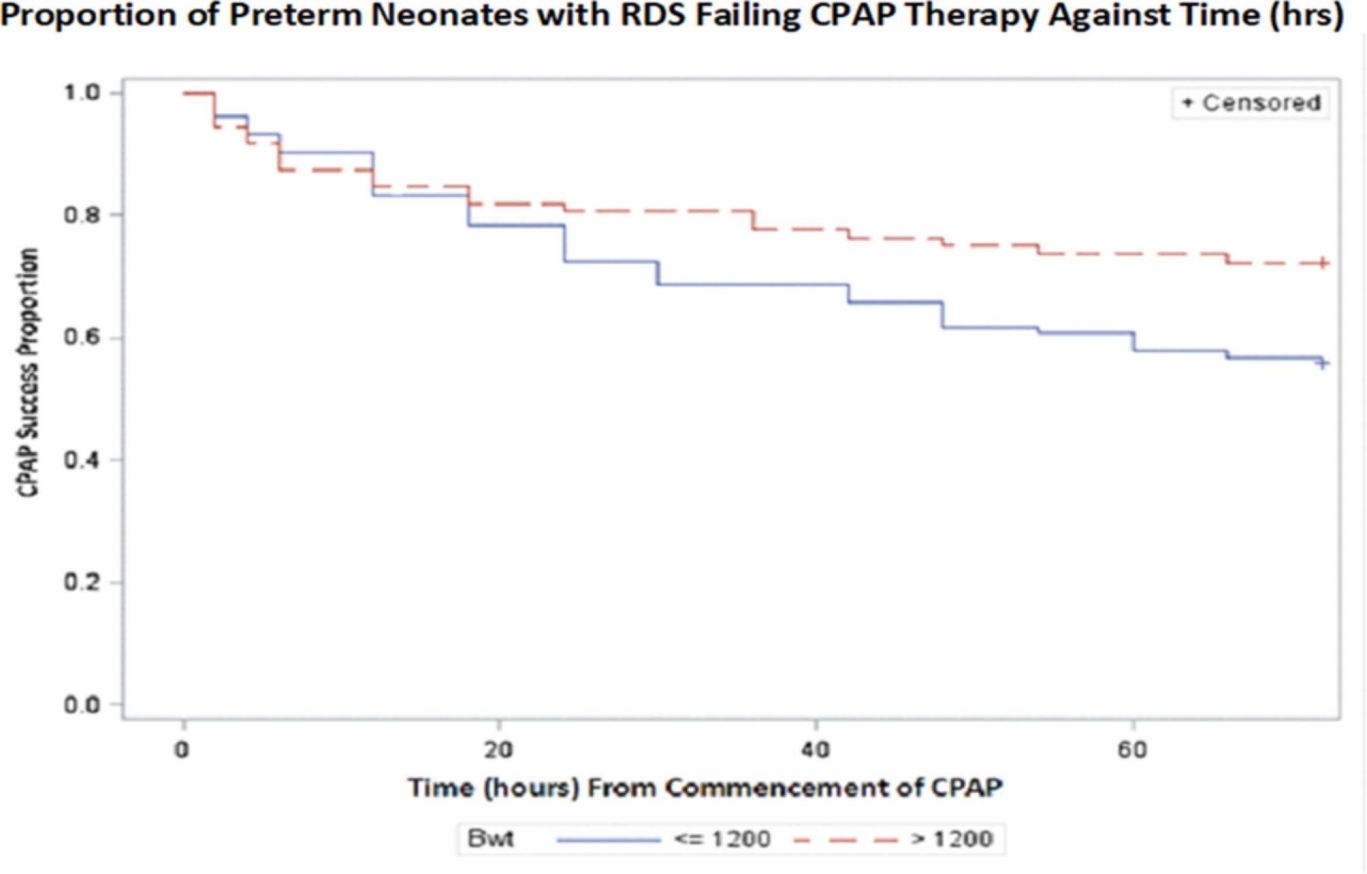
Kaplan Meier curve showing time to CPAP failure

The majority of study infants who failed CPAP therapy did so in the initial 24 h. Figure 3 shows the time to CPAP failure for infants ≤ 1200 g compared to those who were > 1200 g.

## Discussion

Our study demonstrated a CPAP failure rate of 37% among infants of less than 34 weeks gestational age and weighing less than 1500 g. This prevalence is higher than that found in studies done in Australia and New Zealand, Poland, and India that found failure rates of 25%, 28% and 14% respectively [16, 17, 19]. The lower rates could be attributed to the standard of care in all these studies which included antenatal corticosteroids, early initiation of CPAP in the delivery room and early rescue surfactant therapy.

The failure rate found in our study is in line with findings from a systematic review looking at the safety and efficacy of CPAP in low- and middle-income countries, where CPAP failure rates of between 20 and 40% were reported [15] although our failure rate may actually be an underestimation considering the fact that we used a very high fraction of inhaled oxygen of ≥ 50% to classify our study infants as failure. The CPAP failure threshold varies from one institution to another and it is one subject that needs an urgent consensus especially for low resource settings.

The only factor we found to be associated with CPAP failure was birth weight of ≤ 1200 g (OR 2.05 CI 1.07–3.92, p 0.029), this finding is in- line with findings from other studies [17, 19] although in the multivariate analysis it was not independently associated, we attribute this finding to our small sample size. The majority of our study infants (81.4%) with weight ≤ 1200 g were of gestational age ≤ 30weeks. The severity of RDS is inversely related to gestational age [3, 4, 9] which explains why the close to half (44.1%) of our study infants weighing ≤ 1200 g failed CPAP compared to 27.8% of those weight > 1200 g.

Different studies have described early initiation of CPAP to be associated with reduced need for surfactant, mechanical ventilation as well as better survival [14, 17, 25]. This trend was not observed in our study since our centre does not provide CPAP therapy electively. Study infants who needed CPAP therapy within the first 6 h (38/95) had a failure rate of (40%) compared to those who needed after 6 h 26/73(35.6%), this difference was higher among those weighing ≤ 1200 g (51.9% vs. 36.9%), this could be because infants who needed CPAP earlier had much severe disease.

Although early initiation of CPAP for preterm neonates with RDS has been associated with better outcomes [14, 17], the role of prophylactic CPAP for preterm neonates at risk for RDS has not yet been explored [25]. We observed a significant rate of CPAP failure 27/52(51.9%) among preterm neonates ≤ 1200 g who needed CPAP to be initiated within the first 6 h, whether these infants would have had a better outcome had CPAP been initiated before they became symptomatic or not is an area that needs more studies especially in low resource settings where both CPAP and surfactant are a scarce resource.

The use of antenatal corticosteroids is associated with promotion of lung maturation and surfactant production hence better outcome for preterm neonates [6, 26]. In our study only 44/172 (25.5%) of the mothers received steroids; the only steroid given was dexamethasone and only 2 receiving the complete course. This could explain why we did not see any impact of antenatal corticosteroids on CPAP failure in this study.

Among our study infants we observed a high mortality among those who failed CPAP therapy 22/65 (33.8%) compared to 14/109 (12.8%) who did not. This finding is in line with finding from other studies that have described higher morbidity including prolonged hospital stay, bronchopulmonary dysplasia (BPD) as well as higher mortality among preterm neonates who fail CPAP therapy [16, 17, 19, 20]. We did not follow up the 43(66.2%) of our study infants who failed CPAP but remained alive beyond 72 h. These made up 43/174(24.7%) of our study population who remained at high risk for BPD and retinopathy of prematurity.

The majority of study infants who failed CPAP did so in the first 24 h (Fig. 3), this finding is not surprising considering that the majority of our study infants were ≤ 30weeks of gestation and were not steroid mature. Most centres use fraction of inspired oxygen (FiO_2_) cutoff of 35% in the first 2 h to escalate care hence reducing failure rate [16, 19]. Our centre did not have escalation protocols in place during the study period which is typical in setting where there is scarce surfactant. There is urgent need for consensus definition of CPAP failure for resource limited settings to inform the gap in RDS escalation care.

## Conclusion and recommendations

Our study has shown concerningly high rates of CPAP failure as well as mortality of preterm neonates with RDS on CPAP. Neonates weighing ≤ 1200 g and requiring CPAP within 6 h of birth are more likely to fail. Implementing a protocol of early escalation of care for these high-risk infants including surfactant administration may improve outcomes. Further studies are needed to establish methods to minimize CPAP failure in low resource settings.

### Limitations

We did not obtain chest x-ray on study infants failing CPAP hence we cannot clearly explain reason for failure whether it was severe RDS or complications associated with CPAP such as air leaks.

We did not investigate for other factors that may mimic CPAP failure such as intraventricular haemorrhage and hemodynamically significant patent ductus arteriosus.

With fear of air leaks and lack of easy access to chest x-ray our unit uses maximum PEEP of 6 cmH_2_O on the CPAP this may have contribute to the observed high CPAP failure rate.

Our study was conducted during COVID-19 outbreak and this may have affected the overall study dynamics. We were not able to test for COVID-19 at that time.

We did not recruit the desired number of study participants, this could have affected our finding especially on determination of factors associated with CPAP failure.

## Data Availability

The datasets used and/or analyzed during the current study are available from the corresponding author on reasonable request.

## List of abbreviations

ANCs: Antenatal corticosteroids
BPD: Bronchopulmonary dysplasia
COVID19: Coronavirus disease 19
CPAP: Continuous Positive Airway Pressure
LMICs: Low- and Middle-Income Countries
MUHAS: Muhimbili University of Health and Allied Sciences
NBS: New Ballard Score
PEEP: Peak End Expiratory Pressure
RDS: Respiratory Distress Syndrome
SAS: Silverman Andersen Score
